# Disruptions in a cluster of computationally identified enhancers near *FOXC1* and *GMDS* may influence brain development

**DOI:** 10.1007/s10048-015-0458-9

**Published:** 2015-09-17

**Authors:** Genevieve D. E. Haliburton, Gabriel L. McKinsey, Katherine S. Pollard

**Affiliations:** Gladstone Institutes, 1650 Owens Street, San Francisco, CA 94158 USA; Institute for Human Genetics, University of California, San Francisco, San Francisco, CA USA; Department of Anatomy, University of California, San Francisco, San Francisco, CA USA; Division of Biostatistics, University of California, San Francisco, San Francisco, CA USA

**Keywords:** Transcription factor, Development, Enhancer, Gene regulation

## Abstract

Regulatory elements are more evolutionarily conserved and provide a larger mutational target than coding regions of the human genome, suggesting that mutations in non-coding regions contribute significantly to development and disease. Using a computational approach to predict gene regulatory enhancers, we found that many known and predicted embryonic enhancers cluster in genomic loci harboring development-associated genes. One of the densest clusters of predicted enhancers in the human genome is near the genes *GMDS* and *FOXC1. GMDS* encodes a short-chain mannose dehydrogenase enzyme involved in the regulation of hindbrain neural migration, and *FOXC1* encodes a developmental transcription factor required for brain, heart, and eye development. We experimentally validate four novel enhancers in this locus and demonstrate that these enhancers show consistent activity during embryonic development in domains that overlap with the expression of *FOXC1* and *GMDS*. These four enhancers contain binding motifs for several transcription factors, including the *ZIC* family of transcription factors. Removal of the ZIC binding sites significantly alters enhancer activity in three of these enhancers, reducing expression in the eye, hindbrain, and limb, suggesting a mechanism whereby *ZIC* family members may transcriptionally regulate *FOXC1* and/or *GMDS* expression. Our findings uncover novel enhancer regions that may control transcription in a topological domain important for embryonic development.

**Electronic supplementary material:**

The online version of this article (doi:10.1007/s10048-015-0458-9) contains supplementary material, which is available to authorized users.

## Introduction

Enhancers are gene regulatory elements—DNA regions that bind transcription factor proteins to control the timing, amplitude, and tissue specificity of gene expression [1]. The transcription factor binding sites within enhancers often have highly specific motifs, and small mutations in these binding sites can reduce or destroy binding affinity between the enhancer and the transcription factor, leading to alterations in gene expression. In a canonical example of a mutation in an enhancer causing a developmental disorder, a single point mutation in a limb-specific enhancer of the *Shh* (Sonic Hedgehog) gene is sufficient to cause preaxial polydactyly [2].

In this study, we demonstrate a computational approach to predict enhancers, named EnhancerFinder [3], which we used to identify a dense cluster of novel enhancers located in a topologically associating domain (TAD) containing the genes *FOXC1* and *GMDS. FOXC1* is a forkhead box transcription factor that is expressed in the mesenchyme of the developing somites, heart, bone, brain, and other tissues. The mouse homolog *Foxc1* has been studied extensively, with *Foxc1*-null mutants being pre- or perinatal lethal. In humans, complete lack of *FOXC1* is also typically pre- or perinatal lethal, and deletions and point mutations in *FOXC1* contribute to eye and brain developmental disorders [4]. *GMDS* is a short-chain mannose dehydrogenase enzyme that catalyzes the production of GDP-fucose from GDP-mannose. Fucosylated glycans are known to regulate cell-cell adhesion, and thus GMDS may play a role in the regulation of cell migration and axonal pathfinding. Recent studies of the zebrafish GMDS mutants *slytherin* and *towhead* have highlighted the role of fucosylation in neural development. *Slytherin* mutants, which have a point mutation in GMDS, have severe hindbrain malformation and motor dysfunction that result from improper migration of hindbrain neural precursors [5]. The *towhead* mutation in zebrafish is less severe, resulting from a mutation of a conserved Trp residue to an Arg, manifesting in the malformation of the vagal nerve nuclei [6].

We tested four of the predicted enhancers in the *FOXC1/GMDS* domain and found that they consistently drive expression in developing embryonic tissues where these genes are also expressed. These enhancers contain computationally predicted binding sites for many transcription factors, including *ZIC* transcription factors (zinc finger of the cerebellum). We showed that removing ZIC binding sites from these enhancers significantly reduces enhancer expression in the hindbrain, eye, and limb, suggesting that ZIC genes may be involved in the transcriptional regulation of *FOXC1* and/or *GMDS*.

## Materials and methods

### Enhancer identification

Enhancers tested in this study were computationally predicted using EnhancerFinder [3], a machine-learning approach that integrates thousands of genetic and epigenetic data sources to predict developmental human enhancers. The machine-learning algorithm in EnhancerFinder is unique compared to other enhancer prediction approaches, as it was trained using 700 in vivo-validated developmental enhancers from the VISTA Enhancer Browser [7]. The algorithm uses sequence motifs and patterns of transcription factor binding and histone modifications to distinguish these validated enhancers from random genomic regions or from sequences that were tested by VISTA but showed no enhancer activity. The resulting 84,301 novel enhancer candidates are predicted to be active in a variety of embryonic tissues.

### Computational analysis of enhancer clusters

For every gene in the human genome (based on an October 2012 download of the UCSC Genome Browser RefGene track), we counted the number of predicted enhancers within 500 kilobases (kb) upstream or downstream of the transcription start site. While it is hard to know which gene an enhancer is actually regulating, we assumed that 1 megabase (Mb) would be a wide enough distance to capture many of the functional gene-enhancer pairs. We ranked genes based on the number of associated enhancers and thereby identified the loci with the densest clusters of enhancer candidates in the human genome.

The GREAT tool [8] was used to annotate the biological functions of the 1321 genes with the densest clusters of predicted enhancers (top 5 % of all human genes). To test if these genes are enriched for particular functions, we used the hypergeometric test, with the default GREAT settings and the “basal plus extension” association rule (proximal 5 kb upstream, 1 kb downstream, plus distal up to 100 kb).

### Selection of enhancers for functional studies

We focused on a dense cluster of enhancers, which is located in the chromosome band 6p25.3 at the genomic coordinates listed below (hg19) and contains 72 enhancers within 1 Mb. Three of the enhancers (CE1–3) are located in the ∼10-kb intergenic region that falls between the neurodevelopmental genes *FOXC1* and *GMDS*. CE4 is in the second intron of *GMDS*, approximately 90 kb from *FOXC1*. CE1–4 range in length from 995 to 1431 bp. DNA for these regions was synthesized rather than amplified via PCR, but the primers shown below have been used in a related study to amplify the same regions [3] (CE1, located at hg19 chr6:1614904-1616335, forward primer AGACCCCTGTTAGTTTCGCT and reverse primer ATTAGCTGATTCCCCGCCAT; CE2, located at chr6:1616341-1617336, forward primer AAATAGCCTCTGTAAAAAGCTTTAGG and reverse primer GACTGACACAGTCTCTTGGTCCT; CE3, located at chr6:1619847-1620844, forward primer GAGTCGAGTCCTCGGAGC and reverse primer TATGACTACGACGGCAGAGG; CE4, located at chr6:1702408-1703763, forward primer CAGTAGCTGGACTCCGACTC and reverse primer ACTTCCACCCAGCACAGAAA). Wild-type enhancer sequences consisted of the human reference genome (hg19) for each region indicated. ZIC-enhancer sequences were comprised of the reference genome sequence with all computationally predicted ZIC binding sties removed. Position weight matrices for ZIC1 and ZIC3 from the April 2011 release of the TRANSFAC database of experimentally derived transcription factor binding motifs were used to identify ZIC binding sites. The consensus sequence of ZIC binding sites is GGGGTGGTC.

### Associating enhancers with genes

To link enhancers to potential target genes, we used published TADs in human embryonic stem cells. TADs are stable features of mammalian genomes that delineate regions of local 3D chromatin interactions, and the boundaries of these domains remain largely consistent across cell types and between species [9]. ‬The topological domain that contains *FOXC1*, *GMDS*, and the predicted enhancers is defined by chromosomal position chr6:1400001-2680000 (6p25.3-6p25.2) (hg19) and spans 1.28 Mb.‬‬‬‬‬‬‬‬

### Transgenic mouse enhancer assay

Mouse enhancer assays were carried out in transient transgenic mouse embryos generated by pronuclear injections of enhancer constructs into FVB embryos. Human DNA sequences were inserted upstream of the Hsp68 minimal promoter and a *lacZ* reporter gene. The embryos were collected and X-gal stained at E11.5. In this assay, the minimal promoter should only activate the *lacZ* reporter gene when the candidate enhancer region effectively recruits and binds transcriptional machinery. Following standard annotation procedures [7], we required that consistent expression patterns be present in three or more embryos to consider the candidate region an enhancer. This assay includes two negative controls: the empty vector injection and PCR-negative embryos of injected constructs. Background expression of these controls is considered in our annotation criteria. Neither of the controls exhibits the expression patterns seen in the CEs. CE1, 2, 3, and 4 all met this annotation criterion. Transgenic mice were generated by Cyagen Biosciences, whose facility meets and often exceeds animal health and welfare guidelines. Animals were euthanized using techniques recommended by the American Veterinary Medical Association. All procedures were carried out in line with Gladstone Institutes and University of California guidelines.

### Identification of upstream regulators of *FOXC1/GMDS*

Genome-wide locations of predicted transcription factor binding sites were generated using the Find Individual Motif Occurrences (FIMO) tool from the MEME suite of bioinformatics tools [10], based on the April 2011 release TRANSFAC database of experimentally derived transcription factor binding motifs [11], with a FIMO score threshold of 10e−5. We intersected these predicted binding sites with the genomic coordinates of the four enhancers using the IntersectBed tool from the BedTools suite of bioinformatics tools [12].

To determine which genes were expressed in the developing brain, we used the fetal brain data from the GNF Atlas2 database [13]. The fetal brain data included in GNF Atlas2 is based on a Clontech pooled sample of normal whole brains from 59 spontaneously aborted male and female Caucasian fetuses, ages 20–33 weeks. All array data was mapped to RefSeq gene names (RefGene track downloaded from the UCSC Genome Browser January 2014). Genes with an expression score greater than or equal to 64 were considered expressed. To assess known gene expression patterns in the developing mouse, we viewed images of in situ hybridizations of E11.5 and E14.5 mouse embryos from the Allen Brain Map (http://developingmouse.brain-map.org/) and the Eurexpress Transcriptome Atlas (http://www.eurexpress.org/ee/). To assess known gene expression in the developing human brain, we viewed RNA-seq data from the BrainSpan Atlas of the Developing Human Brain (http://www.brainspan.org/). Genes with RPKM (reads per kilobase per million) values greater than 1 were considered to be expressed [14].

## Results

EnhancerFinder is a computational tool that predicts developmental enhancers based on positive examples of biologically active developmental enhancers [7] and negative examples from genomic background. This method uses a multiple kernel learner (similar to a support vector machine) and characterizes genomic regions through an integrated profile of a large number of genetic and epigenetic data sources. Using in vivo-validated examples to train EnhancerFinder and integrating hundreds of sequence motifs and functional genomics experiments make this approach more accurate at identifying biologically active enhancers compared to other approaches [3]. For this study, we started with the 84,301 candidate developmental enhancers predicted by EnhancerFinder across the human genome. We then examined the genome-wide distribution of EnhancerFinder’s predicted enhancers and found that they cluster near loci that contain important developmental genes. Since developmentally active genes typically rely on tight regulation to exhibit robust spatio-temporal expression patterns, these enhancers likely play a role in coordinating normal development. Genes with the highest number of nearby enhancers in the human genome (Supplemental Table 1) are enriched for several biological functions related to development including epithelial cell development, arterial development, and dorsal/ventral neural tube patterning.

**Fig. 1:**
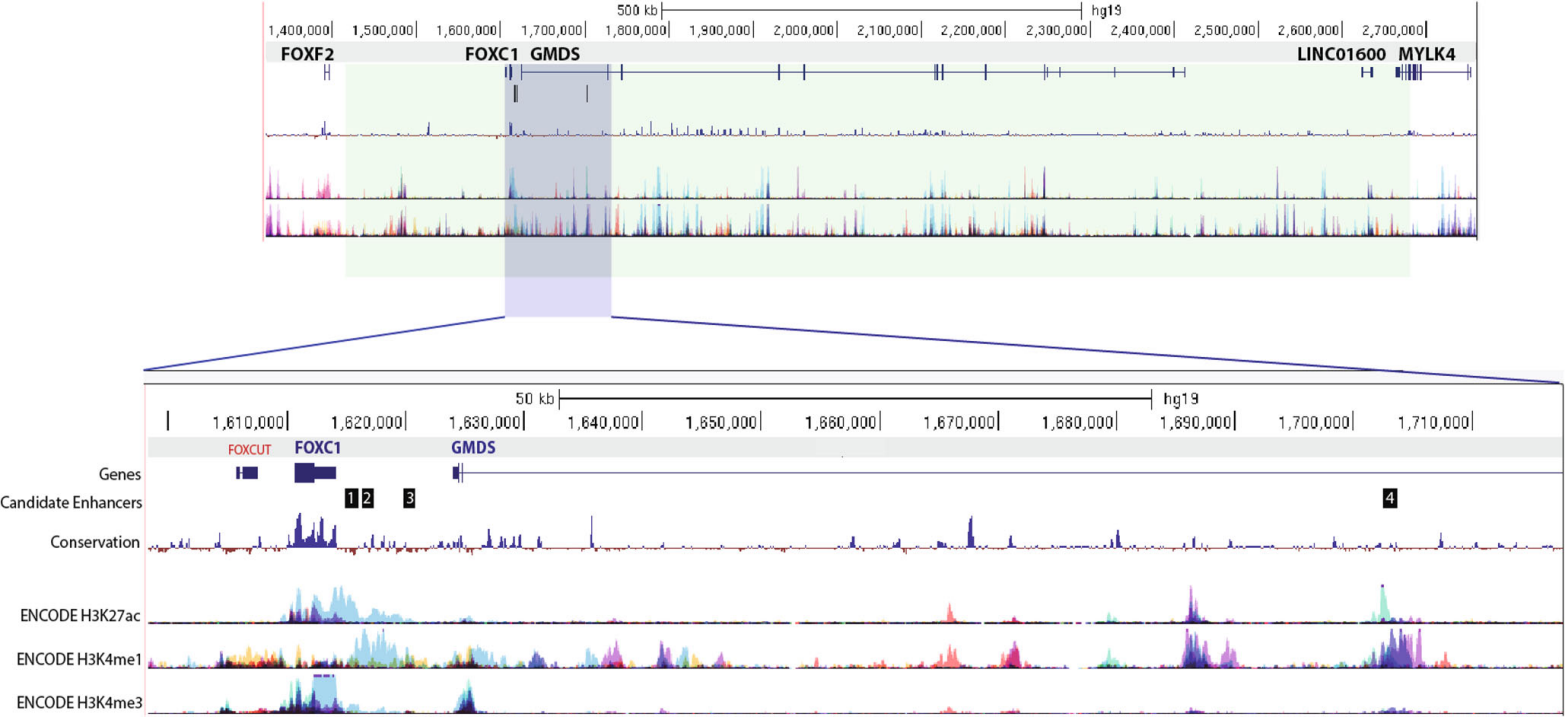
The genomic landscape surrounding *FOXC1* and *GMDS* includes four candidate enhancers tested in this study, shown in a screenshot from the UCSC Genome Browser. The *top panel* shows the entire topological domain that includes *FOXC1* and *GMDS*, highlighted in *green*. The smaller region surrounding the four candidate enhancers is highlighted in *blue* and shown in greater detail in the *lower panel*. The genomic location, genes, and candidate enhancers are shown along with publicly available data tracks of conservation (100 Vertebrates Basewise Conservation by PhyloP) and ENCODE data of enhancer-related histone modifications H3K27ac and H3K4me1, and transcriptional activation-related histone modification H3K4me3 (“layered” view of seven cell lines including GM12878, H1-hESC, HSMM, HUVEC, K562, NHEK, and NHLF)‬‬‬‬‬‬‬‬‬‬‬‬

Many genes essential for normal development fall within clusters of EnhancerFinder’s predicted developmental enhancers. Neighboring genes *FOXC1* and *GMDS* are found in one of the densest enhancer clusters in the genome, with 72 predicted nearby enhancers over a 1-Mb range. These genes and 104 predicted enhancers fall within a single TAD of local chromatin interactions in human embryonic stem cells [9], indicating that these regions have 3D structural interactions during embryonic development. *GMDS* and *FOXC1* are the only two protein-coding genes fully contained within this TAD. Three long non-coding RNA genes are also encoded in this domain, as well as the 3′ end of myosin light-chain kinase *MYLK4*. Boundaries of topological domains are often consistent across cell types and evolution [9], suggesting that the topological domain that contains FOXC1 and GMDS is present in many developing tissues during development (Fig. 1).

**Fig. 2:**
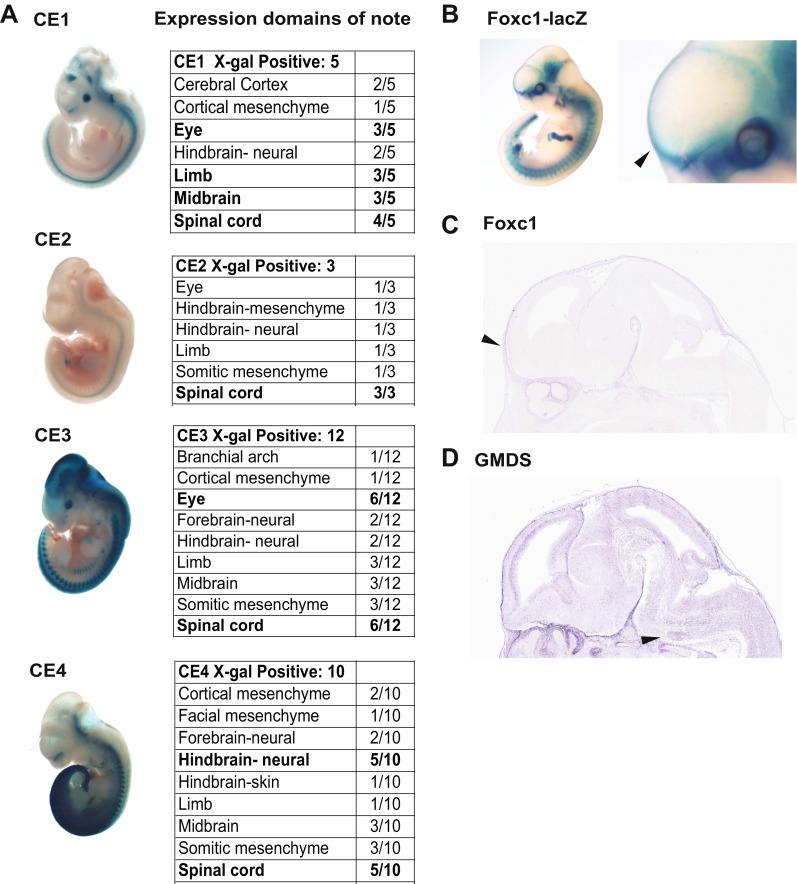
**a** An analysis of four computationally identified enhancers, with annotated tissue expression in the E11.5 mouse embryo. Tissues are noted in *bold* if they have *lacZ* expression half or more of the embryos for a given enhancer. **b** An E11.5 *foxc1-lacZ* embryo, X-gal stained. *Arrowhead* notes cortical mesenchymal expression. E14.5 in situ hybridizations of **c** *foxc1* and **d** gmds in the E14.5 mouse brain. *Arrowhead* notes mesenchymal expression in **c** and hindbrain neural expression in **d**. Data from the Eurexpress transcriptome atlas (http://www.eurexpress.org/ee/)

**Fig. 3:**
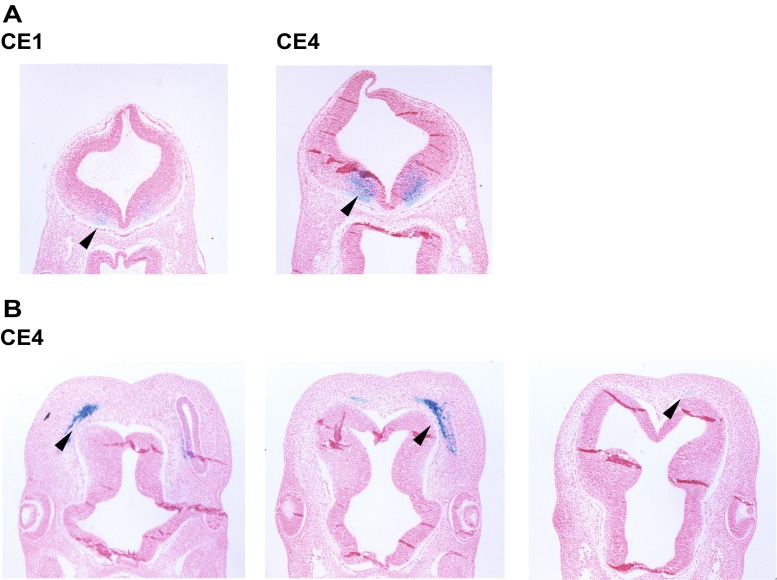
Cryosections of reporter embryos, **a** noting hindbrain neural expression and **b** facial/cortical mesenchyme, see *arrowheads*. Sections are 10 um, horizontally cut, counterstained with FastRed

We tested seven candidate enhancer (CE) regions and validated five novel developmental enhancers near *FOXC1* and *GMDS* using a transgenic mouse enhancer assay. We saw that these five enhancers are active at E11.5 in various embryonic tissues. We chose embryonic day E11.5 as it is an active stage of brain patterning and development and is 1 day prior to when differentiated structures are apparent [15]. Figure 2 shows representative images of CE1–4 from the transgenic mouse assay, as whole embryos, highlighting enhancer activity in different tissues. Associated tables detail the expression patterns of these CEs in the transgenic enhancer assay. A given anatomical region of the embryos was noted in bold when it showed expression in greater than half of the X-gal-positive (+) embryos. For instance, CE1 showed expression in the developing limb in three out of five X-gal-positive embryos (Fig. 2). Additional tissues that showed expression in more than half of the X-gal-positive embryos included the eye (CE1, 2), the spinal cord (CE1–4), and the midbrain (CE1). Expression in the hindbrain neural tube was also seen in embryos for each enhancer, although it was seen most frequently in CE4 (Figs. 2a, 3a). Interestingly, a few embryos had expression in the cortical mesenchyme (CE1, 3, 4) where Foxc1 is expressed endogenously (see Figs. 2b, 3b), although this expression was not consistent across many embryos. CE4 is more likely to be regulating *GMDS* as *GMDS* is expressed in the developing hindbrain neural tube, whereas *Foxc1* is restricted to the surrounding mesenchyme (Fig. 2c, d). CE5 showed consistent expression in the eye (data not shown).

**Fig. 4:**
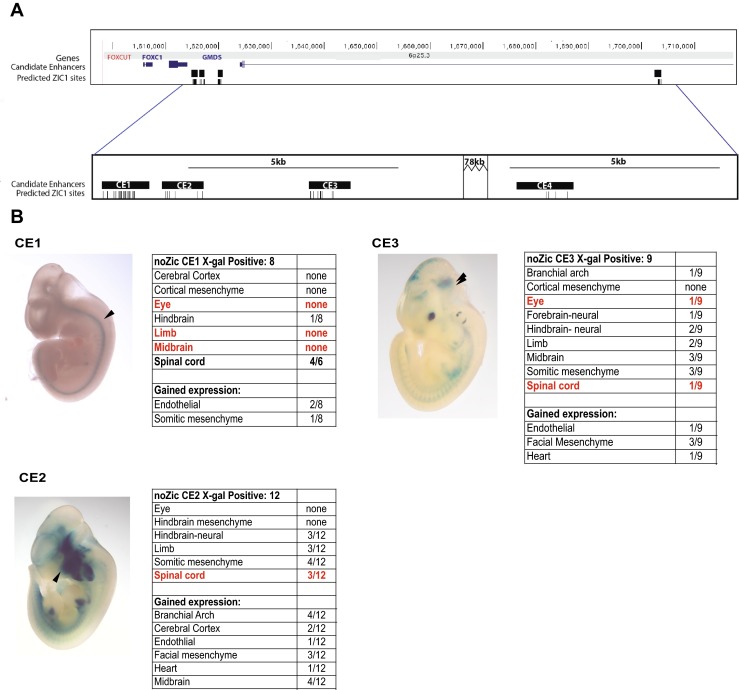
**a** An image from the UCSC Genome Browser showing the enhancer landscape near *FOXC1* and *GMDS*. The *top panel* shows all four CEs. The *lower panel* is zoomed in to the regions immediately surrounding the enhancers, to highlight the ZIC1 binding sites. **b** E11.5 whole-mount images of ZIC mutants CE1–3. CE4 only yielded two X-gal-positive embryos and was thus not used in the analysis. Tissues that have lacZ expression in at least half of the X-gal-positive embryos in WT but not in ZIC enhancers are noted in *red. Arrowheads* mark regions of interest

The four identified CEs contain binding motifs for hundreds of transcription factors. To better understand the region’s transcriptional regulation in brain development, we filtered this list to include transcription factors and co-factors expressed in the developing brain that have at least 12 predicted binding motifs in at least one of the enhancers. We identified a large number of potential transcriptional regulators, widening the number of potential transcriptional regulators of FOXC1/GMDS expression. The neurodevelopmental transcription factors ZIC1 and ZIC3 are amongst the genes with the highest numbers of predicted binding motifs in the CEs (Table 1). Figure 4a shows the enhancer landscape near *FOXC1/GMDS* and the location of predicted ZIC binding sites. ZIC genes, including Zic1 and Zic4, are known to be active in the developing hindbrain [16], suggesting that they may regulate *GMDS* hindbrain expression.‬‬‬‬‬‬‬‬‬‬‬‬‬

**Table 1:** Transcription factors with more than 12 predicted binding motifs found in CEs

Transcription factor name	Total in all CEs	CE1	CE2	CE3	CE4
ESR1	130	58	12	53	7
ELK1	75	29	11	17	18
ZIC3	71	37	10	11	13
PAX5	66	29	2	23	12
DEAF1	62	31	5	19	7
SP1	56	9	6	38	3
PAX3	50	26	3	12	9
MYF6	48	16	7	18	7
ZIC1	48	26	6	12	4
SMAD4	41	15	5	14	7
FOXO1	40	15	15	–	10
SOX13	39	13	6	15	5
EGR1	38	10	5	19	4
MYB	38	8	11	6	13
GATA1	37	3	3	2	29
E2F1	33	14	1	17	1
ATF6	32	11	7	6	8
TBP	31	6	7	6	12
ISL2	30	6	8	2	14
YY1	30	1	18	3	8
PBX1	27	2	15	4	6
CAD	24	7	12	3	2
CDX1	23	6	14	2	1
ELF1	23	6	5	6	6
ELF5	23	9	6	5	3
IK	23	9	3	8	3
IRF3	23	7	9	4	3
SP2	23	6	2	14	1
PAX4	22	12	4	3	3
HNF4A	21	6	8	1	6
IRF4	21	12	–	4	5
MAFB	21	6	7	2	6
SOX9	21	4	11	2	4
ARID3A	20	1	8	1	10
CTCF	20	15	–	3	2
PAX6	20	5	4	6	5
SMAD3	20	4	5	5	6
CEBPA	19	6	7	1	5
FOXO3	19	4	9	–	6
GATA2	19	2	1	1	15
IRF6	19	11	1	4	3
FOXJ1	18	6	5	2	5
PAX8	18	4	5	2	7
TTF1	18	7	5	4	2
USF2	18	8	2	4	4
VDR	18	6	3	3	6
AR	17	8	3	4	2
CEBPB	17	2	7	1	7
SPIB	17	6	–	4	7
E2F3	16	6	–	10	–
POU5F1	16	2	5	–	9
SP3	16	7	4	5	–
ARID5A	15	2	6	1	6
ESRRA	15	5	4	–	6
RXRA	15	6	5	–	4
SP100	15	1	3	8	3
SRY	15	1	12	–	2
TBX5	15	6	3	2	4
GATA3	14	4	1	–	9
LEF1	14	5	5	4	–
NF1	14	2	6	4	2
ZBTB4	14	5	–	2	7
EGR2	13	4	3	6	–
PPARA	13	2	5	3	3
GLIS2	12	2	6	–	4
MAFA	12	2	3	1	6
MAX	12	6	3	3	–
SF1	12	6	–	3	3
SOX2	12	3	4	–	5

For each transcription factor, the total number of predicted binding sites is shown along with the number of predicted binding sites in each CE. These genes are all expressed in the fetal brain (GNF Atlas2 [13] pooled sample of fetal whole brain)

To further investigate the role of ZIC proteins in the regulation of our identified enhancers, we made mutant enhancer constructs that lacked ZIC binding sites. We then used our transgenic mouse enhancer assay to test whether removing these sites affects expression noted in Fig. 2. We found diminished neural expression in three of the four brain CEs (Fig. 4). For the mutant CE4 construct, we did not recover enough embryos to make substantive conclusions (only two X-gal-positive embryos were recovered). Of note, many regions where at least half of the X-gal-positive embryos had expression in with the wild-type (WT) enhancer no longer had robust expression. This included expression in the eye (CE1 and 2), limb (CE1), spinal cord (CE2 and 3), and midbrain (CE1). Also, a number of regions gained expression that was not seen in the WT embryos, including the branchial arch and facial mesenchyme. While mouse ZIC1 or 3 do not appear to be expressed in these structures, ZIC2 is expressed in the developing upper and lower jaws (Figure S1). Thus, ZIC2 may be inhibiting craniofacial expression in the WT context and the lack of ZIC binding sites in the mutant enhancers may lead to disinhibition and activation of expression in craniofacial tissues.

Although the CEs are located very close to *FOXC1* and *GMDS*, they may also regulate other nearby genes. *FOXF2* and *FOXQ1* are located 215 and 300 kb, respectively, upstream of *FOXC1* and the CE cluster. These genes are expressed in many of the same mouse tissues as the CEs (as seen in in situ images of E11.5 mouse embryos from the Allen Brain Map). Although these other potential target genes are not in the same TAD as the CEs in human embryonic stem cells, the genomic distance separating them from the CE cluster is well within the range of enhancer function. Together, *FOXC1*, *GMDS*, *FOXF2*, and *FOXQ1* may represent loci for enhancer-driven changes in gene expression that could affect the developing embryo.

## Discussion

Here, we have applied a novel computational approach for discovering enhancers, called EnhancerFinder. We found that many enhancers cluster around developmentally relevant genes, such as transcription factors that are necessary for controlling cell identity. By identifying novel enhancers, EnhancerFinder provides new avenues of investigation regarding the regulation of gene expression during embryonic development. We characterize a cluster of predicted enhancers around the genes *FOXC1* and *GMDS*, both of which are known to regulate diverse developmental processes in multiple tissues. We found that these enhancers are contained within a single TAD, suggesting a common underlying structural constraint that may link these enhancers to *FOXC1* and *GMDS*. With the large number of EnhancerFinder-predicted regions that cluster within this domain, the *FOXC1* and *GMDS* locus appears to be densely packed with regulatory elements, meriting future investigation.

Using an embryonic mouse transgenic assay, we show that a set of novel enhancers drives expression in many of the regions that express *FOXC1* and/or *GMDS*. We also provide sequence-based evidence that ZIC protein family members such as ZIC1 are likely candidate regulators of expression from these enhancers. To support this, we show that the tissue-specific activity of these enhancers is dependent upon the presence of ZIC transcription factor binding motifs in the enhancers.

A number of developmental processes are regulated by *GMDS* and *FOXC1. GMDS* controls the production of fucosylated glycans, which in turn regulates cell migration and adhesion, as well as the formation of axonal projections and synapses [4, 5, 17]. Notch signaling is also regulated via the fucosylation of EGF-like repeats in the Notch receptor, the disruption of which was shown to contribute, at least in part, to the *slytherin GMDS* mutant phenotype [5]. *FOXC1* controls the development of a number of tissues, including the mesenchyme that surrounds the nervous system, as well as the somatic mesenchyme, heart, kidney, and eye. *FOXC1* mutations in humans result in developmental malformations of the cerebellum and eye, Axenfeld-Rieger syndrome, and Dandy-Walker malformation [4]. These disorders likely result from defective meningeal development and disrupted mesenchymal-neuroepithelial interactions during embryonic development.

The specific role of regulatory elements in the *FOXC1/GMDS* locus during brain development is unclear. Specifically, it is unknown what role non-coding mutations around the *FOXC1/GMDS* locus might play in developmental disorders such as Dandy-Walker syndrome. A small number of the transgenic embryos tested in this study showed mesenchymal enhancer expression suggestive of the *FOXC1-*like expression, although the number was too few to make any definitive conclusions. Neuronal expression was seen more consistently, similar to neuronal *GMDS* expression in the embryonic brain. Given the role of *GMDS* in hindbrain development in zebrafish [5, 6], it is possible that mutations in enhancers surrounding the FOXC1/GMDS locus may disrupt GMDS expression and thus cerebellar development. Also, ZIC genes in humans and in mice have been shown to be involved in Dandy-Walker cerebellar malformations [16, 18], which is interesting given the particularly high number of ZIC binding motifs in enhancers near FOXC1 and GMDS. Thus, these novel enhancers provide mechanistic insight into means by which these two genes are regulated in a variety of tissues in the developing embryo. The initial characterization of the FOXC1/GMDS regulatory environment we present here may help inform further areas of research regarding developmental malformations that result from mutations in this locus.

## Electronic supplementary material

Supplemental Figure 1 An E14.5 in situ hybridization of Zic2, from the Eurexpress Transcriptome Atlas. Arrow heads note expression in the developing upper and lower jaws (http://www.eurexpress.org/ee/). (PDF 10390 kb)

Table S1 Genes with the top 5 % highest number of enhancers within a 1-Mb window (500 kb upstream and downstream) of the transcription start site. The genes are listed in alphabetical order along with the number of nearby enhancers. The number of nearby enhancers ranges from 61 to 106. (TXT 14 kb)
