# Supplementary figures and images for: Disruptions in a cluster of computationally identified enhancers near FOXC1 and GMDS may influence brain development
Source: Neurogenetics. 2015 Sep 17;17:1–9. doi: 10.1007/s10048-015-0458-9 (PMC4701781; doi:10.1007/s10048-015-0458-9)

Supp. Fig. 1

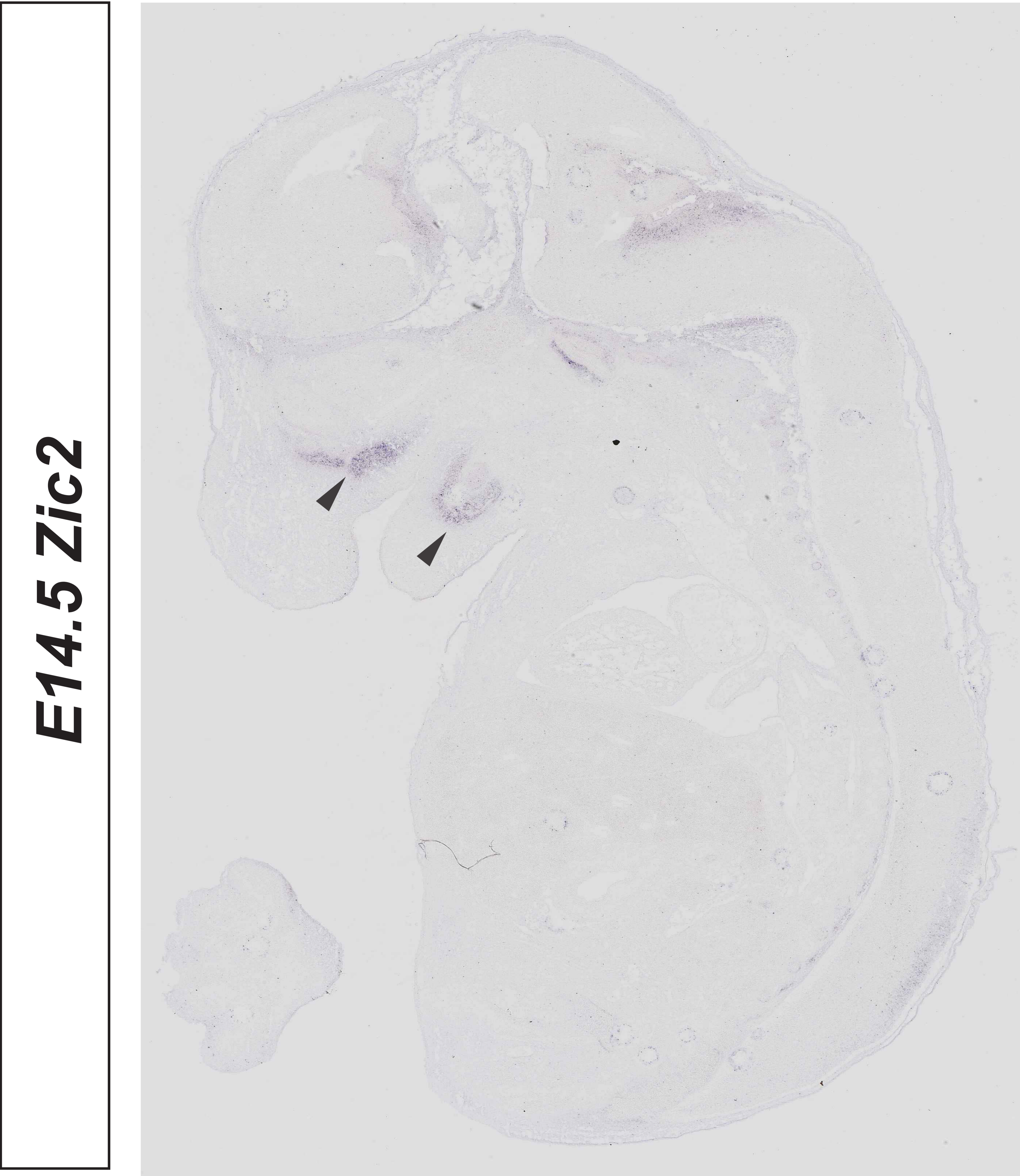

(<http://www.eurexpress.org/ee/>)

Supplement: Supplementary file 1 — An E14.5 in situ hybridization of Zic2, from the Eurexpress Transcriptome Atlas. Arrow heads note expression in the developing upper and lower jaws (http://www.eurexpress.org/ee/). (PDF 10390 kb) [file 10048_2015_458_MOESM1_ESM.pdf]
